# Predicting lncRNA–Protein Interactions by Heterogenous Network Embedding

**DOI:** 10.3389/fgene.2021.814073

**Published:** 2022-02-04

**Authors:** Guoqing Zhao, Pengpai Li, Xu Qiao, Xianhua Han, Zhi-Ping Liu

**Affiliations:** ^1^ Department of Biomedical Engineering, School of Control Science and Engineering, Shandong University, Jinan, China; ^2^ Faculty of Science, Yamaguchi University, Yamaguchi, Japan

**Keywords:** lncRNA–protein interaction, computational method, heterogenous network, network embedding, LncPNet

## Abstract

lncRNA–protein interactions play essential roles in a variety of cellular processes. However, the experimental methods for systematically mapping of lncRNA–protein interactions remain time-consuming and expensive. Therefore, it is urgent to develop reliable computational methods for predicting lncRNA–protein interactions. In this study, we propose a computational method called LncPNet to predict potential lncRNA–protein interactions by embedding an lncRNA–protein heterogenous network. The experimental results indicate that LncPNet achieves promising performance on benchmark datasets extracted from the NPInter database with an accuracy of 0.930 and area under ROC curve (AUC) of 0.971. In addition, we further compare our method with other eight state-of-the-art methods, and the results illustrate that our method achieves superior prediction performance. LncPNet provides an effective method via a new perspective of representing lncRNA–protein heterogenous network, which will greatly benefit the prediction of lncRNA–protein interactions.

## 1 Introduction

The non-coding RNA (ncRNA) plays important roles in biological processes, which can influence human health on various levels (Louro et al., 2009). Existing studies have shown that less than 2% of the human genome can be translated into proteins; while, over 80% of the genome has biochemical functions (Djebali et al., 2012). In addition, over 70% of ncRNAs are lncRNAs (Yang et al., 2014). It is demonstrated that lncRNAs play crucial roles in transcription, splicing gene expression (Ponting et al., 2009; Guttman and Rinn, 2012; Qu and Adelson, 2012; Zhu et al., 2013), and have a close relationship with complex diseases (Mercer et al., 2009; Yang et al., 2015). Therefore, lncRNA is of great importance for understanding the mechanisms of biological processes.

Most of the functions of lncRNA are still unknown. One of the mechanisms is lncRNAs usually function by binding to chaperone proteins (Mercer et al., 2009). Hence, the basis for understanding the functions of lncRNAs is to recognize the interactions between lncRNAs and proteins, which can help understand the mechanism of physiological processes. Experimental methods for identifying protein–RNA interactions include ChiRP, CHART, RIP, RIP-ChIP/Seq, and CLIP (Yang et al., 2015). Since these experimental methods are often time-consuming and expensive, an effective computational method is an alternative way for expanding our knowledge of lncRNA–protein interactions (Liu, 2021).

In recent years, some methods for predicting lncRNA–protein interactions have been developed. Muppirala et al. applied random forest (RF) (Breiman, 2001) and support vector machines (SVMs) (Joachims, 1998) to classify an interaction only via the sequence information of lncRNA and protein (Muppirala et al., 2011). Lncpro was developed for predicting lncRNA–protein associations (Lu et al., 2013) by three types of features based on the Fisher linear discriminant approach, including classical protein secondary structures and hydrogen-bond and van der Waals propensities as well as six types of RNA secondary structures. In 2016, IPMiner was proposed to predict lncRNA–protein interactions from sequences, which employed deep learning and further improved the performance using stacked integration (Pan et al., 2016). Hu et al. introduced a method named HLPI-Ensemble specifically for human lncRNA–protein interactions (Hu et al., 2018). HLPI-Ensemble adopts three methods to extract the features of lncRNA and protein from sequences based on three mainstream machine learning algorithms of SVM, RF, and extreme gradient boosting (XGB) (Chen and Guestrin, 2016). Suresh et al. proposed an approach based on SVM classifiers by integrating sequence and structure features of the lncRNA and protein (Suresh et al., 2015). Zhang et al. combined multiple sequence-based features, lncRNA–lncRNA similarity and protein–protein similarity, and predicted lncRNA–protein interactions by RNA sequences and protein sequences as well as known lncRNA–protein interactions (Zhang et al., 2018b). Li et al. proposed a network-based computational method, which used a random walk with restart based on heterogenous network model (i.e., LPIHN), to infer the lncRNA–protein interactions (Li et al., 2015). Although LPIHN employs the method of network embedding, it does not consider the type of node. Moreover, these ordinary random walks cannot well retain the local and global information of the node from the network. LPLNP was developed for calculating the linear neighborhood similarity in the feature space and transferring it into the interaction space to predict unobserved interactions by a label propagation process (Zhang et al., 2018a). Yi et al. introduced a stacking ensemble-based computational model to predict lncRNA–protein interactions, called RPI-SE, which integrated XGB, SVM, and extremely randomized trees (ExtraTree) (Geurts et al., 2006) algorithms (Yi et al., 2020).

However, there are main drawbacks with the aforementioned methods. First, most of their extracted features for proteins as well as lncRNAs are hand-crafted, which consume much time and require strong domain knowledge. What is more, the previous studies attempt to construct a model to predict the lncRNA–protein interactions of all species. All these may lead to low robustness and overly optimistic predictions.

With the development of machine learning, network representation learning algorithm has become a pressing research task (Cui et al., 2019). In this study, we propose a new lncRNA–protein interactions prediction model called LncPNet based on heterogenous network embedding, which can solve the aforementioned problems in the existing methods. LncPNet is intentionally designed for predicting lncRNA–protein interactions in human, and thus it is trained by human lncRNA–protein interaction data. We apply network embedding to automatically generate features for proteins and lncRNAs. Specifically, a lncRNA–protein heterogenous network is constructed with lncRNA–lncRNA similarity, protein–protein similarity, and lncRNA–protein associations. Then, network embedding extracts, lncRNA features and protein features, are then fed into a SVM classifier to predict lncRNA–protein interactions. Moreover, we compare the performance of LncPNet with the previous models on the same benchmark database. The results demonstrate that LncPNet obtains predictive performance with higher accuracy and robustness.

## 2 Materials and Methods

### 2.1 Framework of LncPNet

**FIGURE 1:**
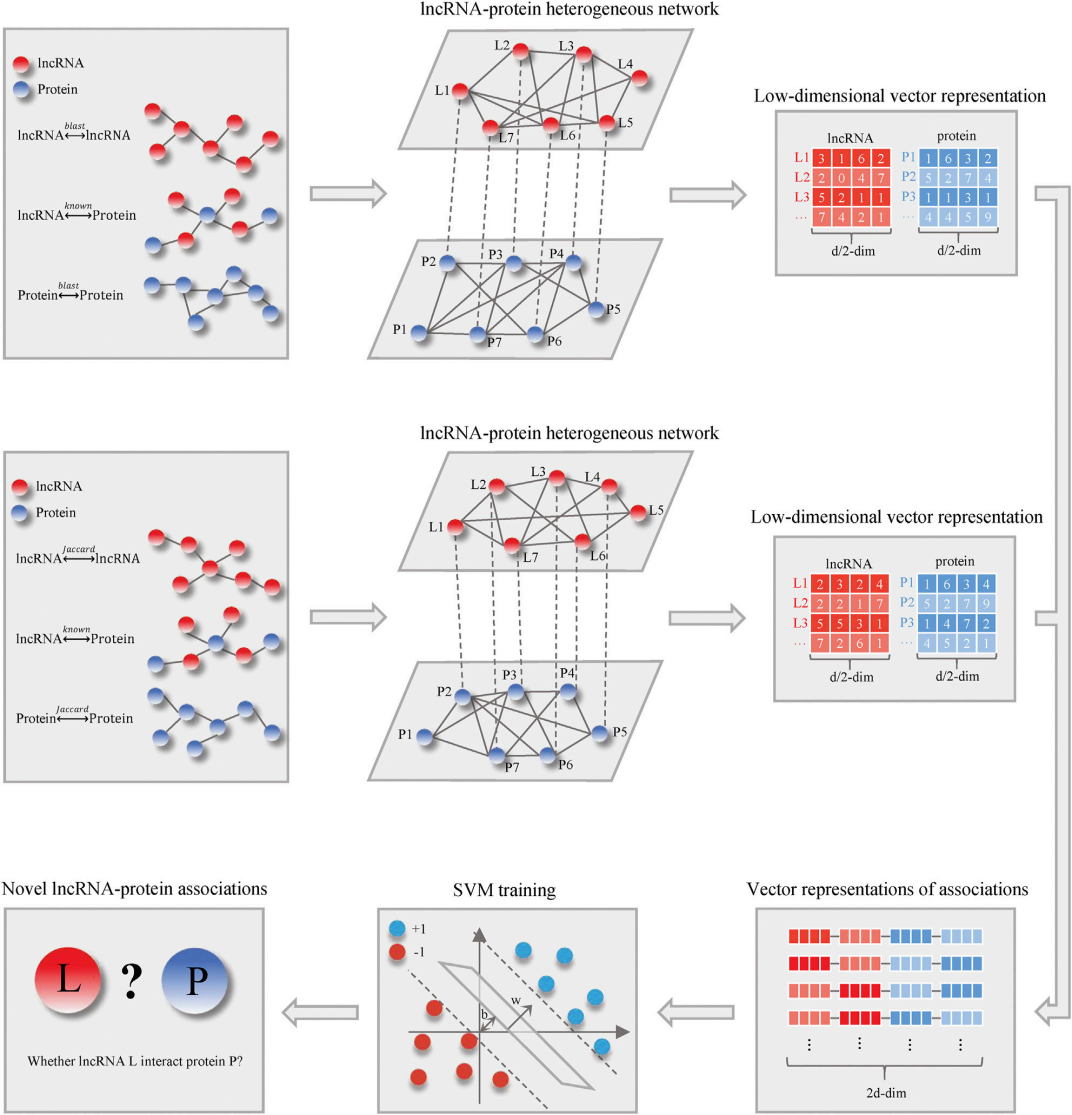
Flowchart of LncPNet.

Figure 1 shows the schematic flowchart of our proposed LncPNet approach for predicting lncRNA–protein interactions based on heterogenous network embedding. The proposed method briefly includes three steps: 1) construction of a heterogenous network based on lncRNA–lncRNA similarity, protein–protein similarity, and known lncRNA–protein interactions; 2) the feature extraction for given lncRNA and protein using network embedding; and 3) training with SVM to predict novel lncRNA–protein associations. More detailed descriptions for each step are given below.

### 2.2 Datasets

In this study, we apply the known lncRNA–protein interaction data from NPInter v2.0 (Yuan et al., 2014) and lncRNA sequence data from NONCODE v6.0 (Zhao et al., 2016) as well as protein sequence data from UniProt (The UniProt Consortium, 2017). NPInter integrates experimentally verified functional interactions between ncRNAs (excluding tRNAs and rRNAs) and other biomolecules (proteins, RNAs, and genomic DNAs). NONCODE aims to present a complete collection and annotation of non-coding RNAs, especially long non-coding RNAs (lncRNAs). The UniProt knowledge base is a large resource of protein sequences and associated detailed annotation. First, we extract the human lncRNA–protein interactions from NPInter, which are filtered by restricting the organism, the type of lncRNAs, and the type of proteins to “Homo,” “ncRNA,” and “protein,” respectively. After data cleaning, we obtain 7,523 experimentally validated human lncRNA–protein interactions, including 3,052 lncRNAs and 212 proteins. Then, we map these lncRNA IDs and protein IDs of NPInter into NONCODE IDs and UniProt IDs, respectively. From these lncRNAs and proteins that we have, we remove lncRNA and protein whose sequence information is unavailable. Finally, we obtain a dataset with 4,578 lncRNA–protein interactions between 2,009 lncRNAs and 78 proteins. In these datasets, only known lncRNA–protein associations (positive samples) are available. To train the classifier, we choose negative samples by a subcellular localization method with empirical tests of other alternatives. So, we randomly choose the same number of samples from all possible negative pairs. Meanwhile, the dataset is randomly divided into two parts, where one part is used for training set and the other is for testing. Among them, the quantity scale of the training set and test set is approximately 9:1, and the procedure is repeated three times.

### 2.3 Construction of a lncRNA–Protein Heterogenous Network

An lncRNA–protein heterogenous network is constructed with lncRNA–lncRNA similarity, protein–protein similarity, and known lncRNA–protein associations. lncRNA–lncRNA similarity and protein–protein similarity are both quantified in two different ways.

#### 2.3.1 Jaccard Similarity

The Jaccard similarity (Bag et al., 2019) is an index used to measure the similarity of two sets. In this study, the Jaccard similarity is employed to calculate lncRNA–lncRNA similarities and protein–protein similarities. We define $L_{\text{i}}=\left\{p_{1},p_{2},...,p_{x}\right\}$ and $P_{j}=\left\{l_{1},l_{2},...,l_{y}\right\}$ as two sets of lncRNA i and protein j , which contain associated proteins of lncRNA i and associated lncRNAs of protein j , respectively. Given two lncRNAs, the similarity between two lncRNAs is defined as follows:

$$J\left(L_{i},L_{j}\right)=\frac{\left|L_{i}\cap L_{j}\right|}{\left|L_{i}\cup L_{j}\right|},$$ (1)

where $L_{i}$ and $L_{j}$ represent lncRNA i and lncRNA j associated proteins sets, respectively.

#### 2.3.2 BLAST Similarity

BLAST is a fundamental and basic local alignment search tool for sequence similarity based on a local optimal alignment strategy (Ye et al., 2006). Essentially, BLAST is a heuristic algorithm. It first breaks the query sequence into sub-segments, called seed words. Furthermore, the seed is compared with the pre-indexed sequence, and the position with the higher continuous score of the seed is selected for further extension by the dynamic programming algorithm. The extension process will also be scored. When the score is below a certain limit, the extension process will be terminated and abandoned. Finally, a series of high-scored sequences are produced. In this study, we establish two local databases for lncRNA and protein. Then, the similarities between every two lncRNAs and every two proteins are calculated via BLAST.

#### 2.3.3 The Heterogenous Network

The lncRNA–lncRNA Jaccard similarity network can be represented using a bipartite graph $G_{11}$ , as follows:

$$G_{11}=\left(L,E_{11},J\right),$$ (2)

where $L=\left\{l_{1},l_{2},...l_{n}\right\}$ represents the set of n lncRNAs, $E_{11}=\left\{e_{1},e_{2},...e_{m}\right\}$ represents sets of edges between vertices, and $l_{i}$ and $l_{j}$ are connected if the Jaccard similarity is more than 0.5.

The lncRNA–lncRNA BLAST similarity network can be represented using a bipartite graph $G_{12}$ , as follows:

$$G_{12}=\left(L,E_{12},B\right),$$ (3)

where $L=\left\{l_{1},l_{2},...l_{n}\right\}$ represents the set of n lncRNAs, $E_{12}=\left\{e_{1},e_{2},...e_{m}\right\}$ represents sets of edges between vertices, and $l_{i}$ and $l_{j}$ are connected if the BLAST similarity *e*-value is less than 0.001.

Similarly, two bipartite graphs $G_{21}$ and $G_{22}$ represent protein–protein similarities as follows:

$$G_{21}=\left(P,E_{21},J\right);$$ (4)

$$G_{22}=\left(P,E_{22},B\right),$$ (5)

where $P=\left\{p_{1},p_{2},...p_{n}\right\}$ represents the set of n proteins, $E_{21}=\left\{e_{1},e_{2},...e_{m}\right\}$ and $E_{22}=\left\{e_{1},e_{2},...e_{m}\right\}$ represent sets of edges between vertices, and $P_{i}$ and $P_{j}$ are connected if their Jaccard similarity is more than 0 and the BLAST similarity *e*-value is less than 0.01.

Then, we construct two heterogenous networks. Among them, one is by known lncRNA–protein interactions, lncRNA–lncRNA similarities, and protein–protein similarities calculated with the Jaccard similarity. The other is by known lncRNA–protein interactions, lncRNA–lncRNA similarities, and protein-protein similarities calculated with BLAST similarity.

### 2.4 Heterogenous Network Embedding

Network embedding can use less information to represent nodes as dense- and low-dimensional vectors and has been rapidly developed and applied recently (Cao et al., 2016; Hamilton et al., 2018; Veličković et al., 2018; Zhang et al., 2020). According to the heterogenous network constructed previously, we employ network embedding to learn the low-dimensional latent representations based on the structural and semantic properties of the lncRNA–protein heterogenous network, which are able to characterize the lncRNA–protein associations. In LncPNet, we adopt the metapath2vec method (Dong et al., 2017) for network embedding because it takes better account of the type of nodes, which is suitable for representing the heterogenous network. Generally, metapath2vec can be divided into two steps. First, we employ meta**-**path-based random walks to generate paths that can capture both the semantic and structural correlations between different types of nodes and then facilitate the transformation of heterogenous network structures into metapath2vec′s skip-grams.

In detail, a meta-path scheme φ from $V_{1}$ to $V_{l}$ is defined as the form of $V_{1}\overset{R_{1}}{\to }V_{2}\overset{R_{2}}{\to }...V_{t}\overset{R_{t}}{\to }V_{t+1}...\overset{R_{l-1}}{\to }V_{l}$ , where $R=R_{1}\circ R_{2}\circ ...\circ R_{l-1}$ is defined as the composite relations between node types $V_{1}$ and $V_{l}$ . In this study, we define “LPLPL” and “LLPPLL” metapaths, in which “LPLPL” represents two lncRNAs interact via a protein and similarly for “LLPPLL”. For the heterogenous network G(V,E) and metapath $V_{1}\overset{R_{1}}{\to }V_{2}\overset{R_{2}}{\to }...V_{t}\overset{R_{t}}{\to }V_{t+1}...\overset{R_{l-1}}{\to }V_{l}$ , the transition probability at step i is defined as follows (Yang et al., 2019):

$$p\left(v^{i+1}|v_{k}^{i},\varphi \right)=\left\{ \begin{array}{l} \frac{1}{\left|N_{j}\left(v_{k}^{i}\right)\right|},\left(v^{i+1},v_{k}^{i}\right)\in E,\phi \left(v^{i+1}\right)=j\\ 0,otherwise, \end{array}\right.,$$ (6)

where $v_{j}$ and $v_{k}$ , respectively, denote the jth and kth node type in the path φ , $N_{j}\left(v_{k}\right)$ denotes the neighborhood of node $v_{k}^{j}$ with respect to the jth node type, and ϕ(v) is a constraint function to make sure the node type of node v to be type j . In order to avoid the disclosure of the test set information, we remove the associations between lncRNA and protein in the test set when the metapath is generated. Then, skip-gram learns effective node representations for a heterogenous network G(V,E) by maximizing the probability of having the heterogenous context.

LncPNet employs metapath2vec on the aforementioned two heterogenous networks to produce a 1 × 64 feature vector for every vertex. Moreover, we splice the two feature vectors of every lncRNA to obtain a 1 × 128 feature vector, which is the same to every protein encoded.

### 2.5 Prediction of lncRNA–Protein Interactions

With vector representations of lncRNA–protein associations as inputs, which of dimensionality is 1 × 256, SVM is trained to predict whether an lncRNA interacts with a protein. In particular, our training set and test set are pre-divided, and we conduct the procedure three times. What is more, we choose radial basic function (RBF) as the SVM kernel function.

### 2.6 Performance Evaluation

Precision (PRE), recall (REC), specificity (SPE), accuracy (ACC), Matthew’s correlation coefficient (MCC), and F1-score are the most common classification model evaluation indicators. They can be defined as (Sokolova et al., 2006):

$$PRE=\frac{TP}{TP+FP};$$ (7)

$$REC=\frac{TP}{TP+FN};$$ (8)

$$SPE=\frac{TN}{FP+TN};$$ (9)

$$ACC=\frac{TP+TN}{TP+TN+FP+FN};$$ (10)

$$MCC=\frac{TP\times TN-FP\times FN}{\sqrt{\left(TP+FP\right)\left(TP+FN\right)\left(TN+FP\right)\left(TN+FN\right)}};$$ (11)

$$F1=\frac{2\times precision\times recall}{precision+recall},$$ (12)

where TP , FP , TN , and FN is the number of true positives, false positives, true negatives, and false negatives, respectively.

## 3 Results and Discussion

### 3.1 Performance of LncPNet

**FIGURE 2:**
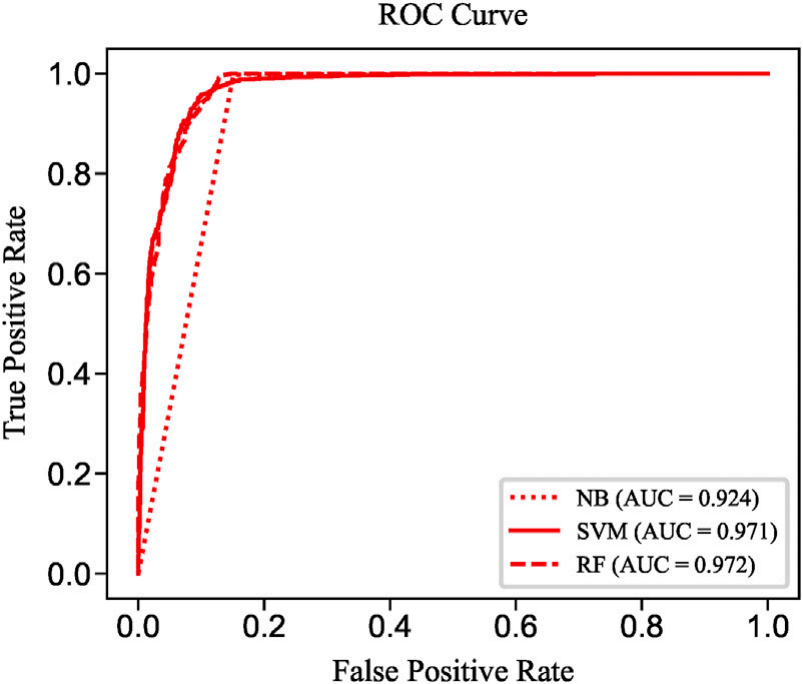
ROC curves of SVM, RF, and NB.

**FIGURE 3:**
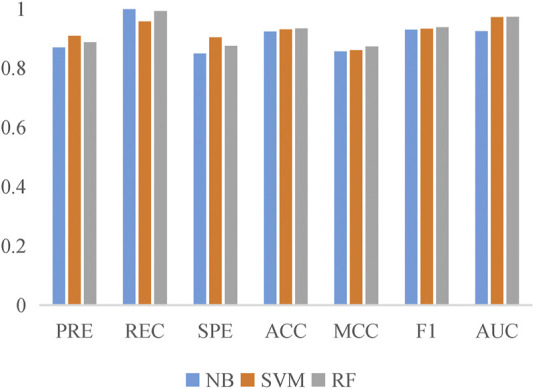
Histogram of the six evaluation criteria achieved by SVM, RF, and NB models.

To evaluate the prediction performance of LncPNet, we test RF (Breiman, 2001), naive Bayesian (NB) (Elkan, 1997), and SVM (Joachims, 1998) classifiers. As shown in Figure 2, SVM achieves the AUC of 0.971 on the NPInter v2.0 dataset. It increases by 4.7% over NB with the AUC of 0.924 and decreases by 0.1% over RF with the AUC of 0.972. But from Figure 3, SVM has comparable performance with RF. Thus, we choose SVM as our classifier implemented in LncPNet. What is more, we test different negative samples producing approaches on this model. Finally, LncPNet employs the SVM classifier to train the model and adopts the subcellular localization method to produce negative samples. For comparison study, we evaluate the performance of CF (Sarwar et al., 2001), RWR (Köhler et al., 2008), LPBNI (Ge et al., 2016), SFPEL-LPI (Zhang et al., 2018b), LPIHN (Li et al., 2015), LPLNP (Zhang et al., 2018a), RPI-SE (Yi et al., 2020), and IPMiner (Pan et al., 2016) on NPInter v2.0. Meanwhile, the performance of different sub-models has also been identified. In order to evaluate the performance of these methods comprehensively, we employ the ACC, PRE, REC, SPE, MCC, AUC, and F1 as the evaluation metrics. AUC (Huang and Ling, 2005) is the area under the ROC (Fawcett, 2006) curve, which is an evaluation dedicated to the classification model. In LncPNet, the average PRE, REC, SPE, ACC, MCC, F1, and AUC is 0.908, 0.957, 0.903, 0.930, 0.860, 0.932, and 0.971, respectively.

### 3.2 Comparisons With Sub-Models

**FIGURE 4:**
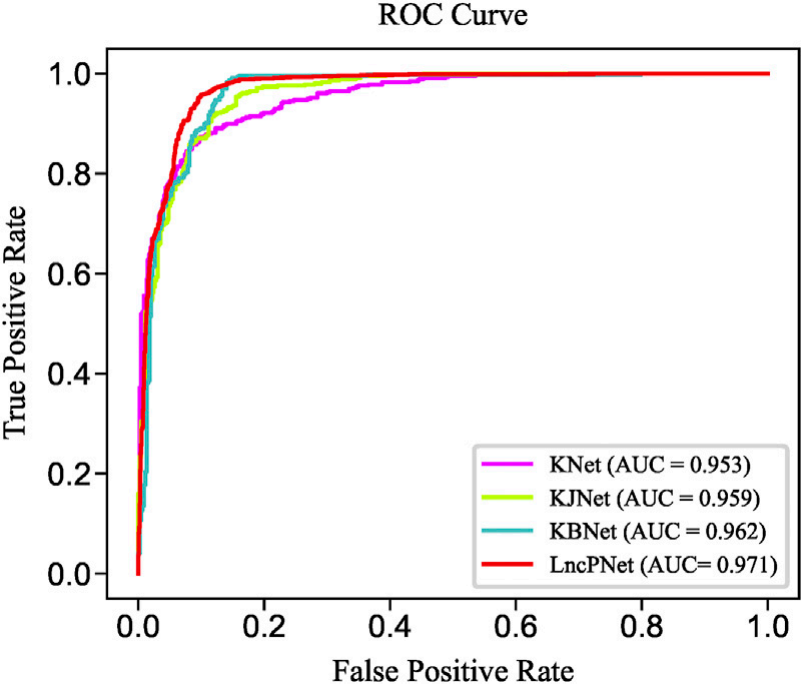
ROC curves of LncPNet and sub-models.

In order to fully evaluate the performance, we compare LncPNet with three sub-models on NPInter v2.0. LncPNet model construction is mainly divided into three steps. Specifically, we construct a heterogenous network with lncRNA–lncRNA similarities, protein–protein similarities, and known lncRNA–protein interactions, where lncRNA–lncRNA similarities and protein–protein similarities are calculated by the Jaccard similarity and BLAST similarity, respectively. Then, a feature vector is generated from the heterogenous network with network embedding (metapath2vec) to characterize a pair of lncRNA and protein. Finally, with the feature vectors with class labels as inputs, SVM is trained to predict potential lncRNA–protein associations. The construction of heterogenous network contains four types of different strategies. In approach 1, only known lncRNA–protein interactions (KNet) are used to construct the network; in approach 2, known lncRNA–protein interactions and Jaccard similarity (KJNet) are used to construct the network; in approach 3, known lncRNA–protein interactions and BLAST similarity (KBNet) are used to construct the network; and in approach 4, known lncRNA–protein interactions, Jaccard similarity, and BLAST similarity (LncPNet) are used to construct the network. Figure 4 shows the ROC curve. Table 1 illustrates the prediction results of different integration strategies on NPInter v2.0. From Table 1, we can find the experiments of LncPNet integrate the advantages of different branch models, achieving better performance than those of sub-models.

**TABLE 1:** Prediction results of LncPNet and sub-models.

Network	PRE	REC	SPE	ACC	MCC	F1	AUC
KNet	0.898	0.873	0.901	0.887	0.774	0.885	0.953
KJNet	0.887	0.914	0.884	0.899	0.799	0.900	0.959
KBNet	0.875	**0.982**	0.859	0.921	0.848	0.925	0.962
LncPNet	**0.908**	0.957	**0.903**	**0.930**	**0.860**	**0.932**	**0.971**

Every bold value means it corresponds to the highest value in the evaluation indicator.

### 3.3 The Strategy of Negative Sampling

Missing negative samples has always been a problem in predicting molecular interactions, which leads to a wide variety of negative sample generation methods. However, few studies have proved how to generate negative samples is the most reliable. In this section, we summarize three commonly used negative sample construction methods. The first one, and also the most popular one, is the random pairing method. Negative samples are randomly sampled from the possible lncRNA–protein pairs except the positive samples. The second one is the method of subcellular localization, which is based on the assumption that the lncRNA and protein that are not in the same subcellular location would not interact with each other. Therefore, proteins and lncRNAs that are not in one organelle are regarded as negative sample pairs. The third one is the network distance method, which calculates the shortest-path distance between each lncRNA and protein in the prior interaction network, and treats the protein and lncRNA that are greater than a certain distance threshold, for e.g., six, as a negative sample pair.

**FIGURE 5:**
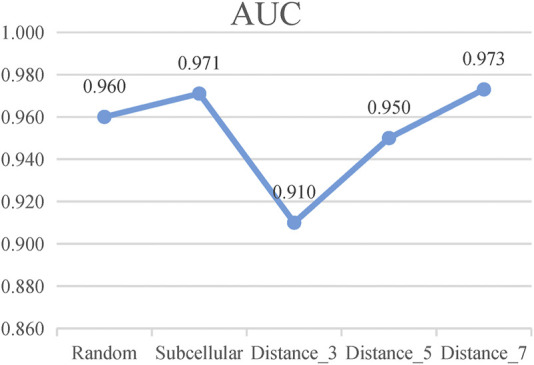
AUC values of Random, Subcellular, “Distance_3,” “Distance_5,” and “Distance_7” (Random, random-pairing method; Subcellular, subcellular localization method).

According to these rules, we further categorize the distance method of selecting negative samples into three types of experiments: 1) “Distance_3”: the negative samples with a distance equal to 3; 2) “Distance_5”: the negative samples with a distance greater than 1 and less than or equal to 5; and 3) “Distance_7”: the negative sample with a distance greater than 1 and less than or equal to 7. To avoid the imbalance problem when training the classifier, we choose negative samples with the same number of positive samples in the experiments. As presented in Figure 5, the subcellular localization method and “Distance_7” achieve a relatively higher value than the random pairing, “Distance_3” and “Distance_5” methods. Meanwhile, in the three distance-based methods, “Distance_3,” “Distance_5,” and “Distance_7”, we find that as the distance of selecting negative sample increases, the AUC value becomes higher. This also validates the rationality of our proposed strategy and the former assumption in selecting negative samples. Table 2 shows that the subcellular localization method achieves the best prediction performance according to the six evaluation metrics. This clearly shows that different negative samples have a concrete impact on the model, and more reliable negative samples will make LncPNet to achieve better prediction results. Thus, we employ the subcellular localization method as our negative sample generation method in LncPNet.

**TABLE 2:** Performance comparison of five negative sample models.

Method	PRE	REC	SPE	ACC	MCC	F1	AUC
Random	0.870	0.946	0.856	0.901	0.808	0.905	0.960
Subcellular	**0.908**	**0.957**	**0.903**	**0.930**	**0.860**	**0.932**	0.971
Distance_3	0.846	0.820	0.851	0.835	0.672	0.833	0.910
Distance_5	0.863	0.915	0.854	0.884	0.771	0.888	0.950
Distance_7	0.905	0.933	**0.903**	0.918	0.837	0.919	**0.973**

Every bold value means it corresponds to the highest value in the evaluation indicator.

### 3.4 Comparison With Other State-Of-The-Art Models

In order to further demonstrate the reliability and robustness of prediction by the LncPNet method, we compare LncPNet with the eight state-of-the-art methods, namely IPMiner, RPI-SE, LPLNP, RWR, CF, SFPEL-LPI, LPBNI, and LPIHN, on the same benchmark of NPInter v2.0. These methods are typical methods that have been proposed in recent years, and they can be divided into three categories:

(1) The first type of method is mainly based on sequence information, structural information, evolutionary knowledge, or physical and chemical properties to mine the distinguishing characteristics of the lncRNA and protein. For example, RPI-SE applied the position weight matrix combined with Legendre moments to obtain protein evolutionary information and k-mer sparse matrix to extract feature of lncRNA sequences. SFPEL-LPI used sequence information to build a feature projection ensemble-learning frame to predict lncRNA–protein interactions.
(2) The second type of method is mainly to use stacked autoencoders to extract high-level hidden features of proteins and lncRNAs. For example, IPMiner extracted raw sequence composition features from lncRNA and protein sequences, high-level features by applying stacked autoencoder, and fine-tuning features using label information, and then a training ensemble strategy such as RF classifier to robustly predict the interactions between lncRNAs and proteins.
(3) The third type of method mainly uses topological information to extract lncRNA and protein features. For example, LPLNP employed a linear neighborhood propagation method, to predict lncRNA–protein interactions. LPBNI used a bipartite network–based method for predicting lncRNA–protein interactions. RWR and CF are also the same type of methods. LPIHN constructed a lncRNA–protein heterogenous network and used a random walk with restart to infer novel lncRNA–protein interactions.

**FIGURE 6:**
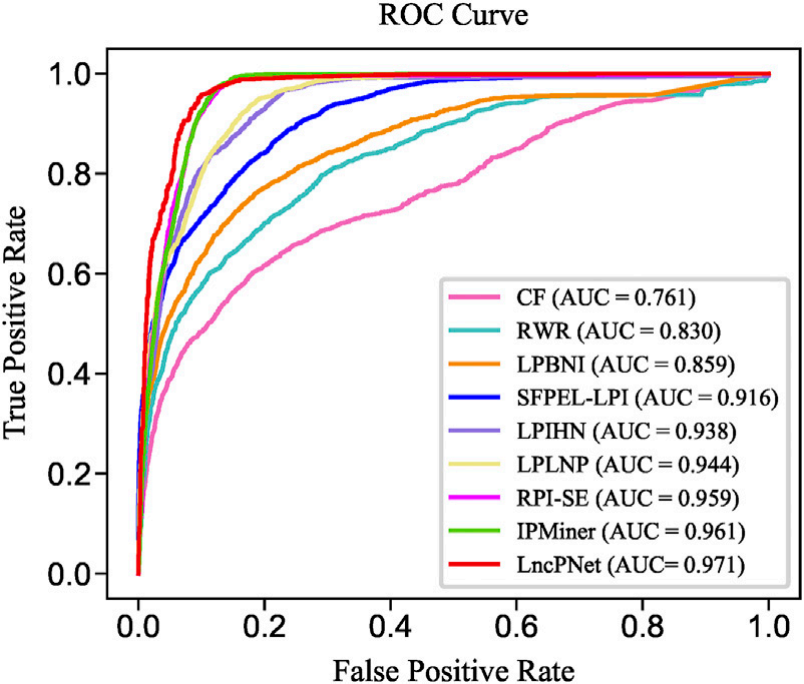
ROC curves of LncPNet and eight comparing methods.

We replicate all these methods on the same dataset for fair comparisons. As shown in Table 3, LncPNet achieves a PRE of 0.908, SPE of 0.903, ACC of 0.930, MCC of 0.860, and F1 of 0.932, which outperform all the other methods. REC is a little worse than the best method, IPMiner. All these performance comparisons indicate that LncPNet has higher reliability in predicting lncRNA–protein interactions. Figure 6 illustrates the ROC curves with AUCs of these methods. The results further demonstrate the effectiveness and advantage of our method, LncPNet. Although we use the heterogenous network with LPIHN, our metapath2vec method takes into account the node type and transition probability simultaneously, which makes it achieves better performance.

**TABLE 3:** Performance comparison of LncPNet and eight available methods.

Method	PRE	REC	SPE	ACC	MCC	F1	AUC
CF	0.583	0.894	0.361	0.627	0.301	0.706	0.761
RWR	0.739	0.798	0.717	0.757	0.517	0.767	0.830
LPBNI	0.740	0.840	0.698	0.769	0.548	0.785	0.859
SFPEL-LPI	0.769	0.920	0.724	0.822	0.657	0.838	0.916
LPIHN	0.807	0.966	0.769	0.867	0.750	0.879	0.938
LPLNP	0.832	0.943	0.810	0.876	0.761	0.884	0.944
RPI-SE	0.877	**0.974**	0.863	0.919	0.843	0.923	0.959
IPMiner	0.886	0.970	0.875	0.922	0.849	0.926	0.961
LncPNet	**0.908**	0.957	**0.903**	**0.930**	**0.860**	**0.932**	**0.971**

Every bold value means it corresponds to the highest value in the evaluation indicator.

### 3.5 Case Study

**FIGURE 7:**
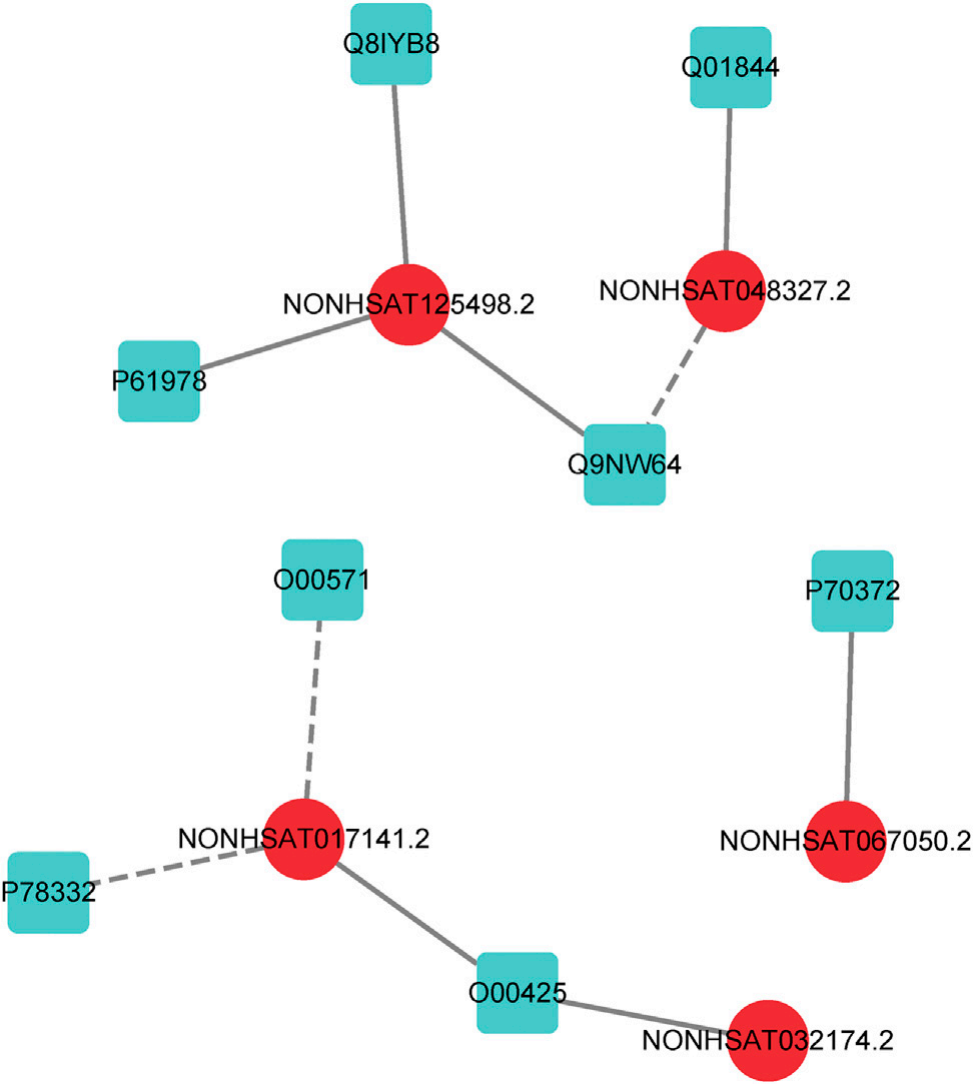
lncRNA–protein network constructed by LncPNet. The lncRNA and proteins are shown in red (circular) and blue (square) nodes, respectively, while the correctly and wrongly predicted interactions are shown as solid and dotted lines, respectively.

In order to further evaluate the reliability of our prediction model, we propose a case study to verify its performance. As mentioned earlier, the dataset we used in LncPNet is NPInter v2.0, and currently NPInter has been updated to NPInter v4.0, which includes some novel lncRNA–protein interaction pairs. We test to predict the new lncRNA–protein interactions confirmed in NPInter v4.0 based on known interactions in NPInter v2.0. Specifically, we predict the 23 pairs of interactions newly discovered in NPInter v4.0 and the generated 23 pairs of negative samples and rank them according to the scores. As shown in Table 4, we list the top ten interactions predicted by LncPNet, in which seven novel interactions are confirmed in the new version of NPInter. Figure 7 illustrates the constructed network diagram. The case study provides more evidence for the effectiveness, flexibility, and extendibility in predicting lncRNA–protein interactions.

**TABLE 4:** Top 10 novel interactions predicted by LncPNet.

Rank	lncRNA	Protein	Whether confirmed
1	NONHSAT032174.2	O00425	Yes
2	NONHSAT017141.2	O00425	Yes
3	NONHSAT125498.2	P61978	Yes
4	NONHSAT048327.2	Q01844	Yes
5	NONHSAT017141.2	O00571	No
6	NONHSAT125498.2	Q9NW64	Yes
7	NONHSAT017141.2	P78332	No
8	NONHSAT048327.2	Q9NW64	No
9	NONHSAT125498.2	Q8IYB8	Yes
10	NONHSAT067050.2	P70372	Yes

## Conclusion

In this study, we proposed LncPNet based on a heterogeneous network embedding method for predicting lncRNA–protein interactions. The experimental results demonstrated that LncPNet achieves high prediction performance on our benchmark dataset and yields better results compared to other methods. As for the lncRNA–protein interaction predictive task is a nonnegative sample problem, we provided a new perspective into network embedding by comparing three kinds of methods for negative sampling. In addition, the case study results further demonstrated the effectiveness of LncPNet. The network embedding method is a general node representing method. The framework of LncPNet can be expanded to other interaction predictive task, such as miRNA–protein interaction prediction and lncRNA–disease interaction prediction.

## Data Availability

The data and code in this study are available at: https://github.com/zpliulab/LncPNet.
